# Short-Term Efficacy of Transcranial Focused Ultrasound to the Hippocampus in Alzheimer’s Disease: A Preliminary Study

**DOI:** 10.3390/jpm12020250

**Published:** 2022-02-09

**Authors:** Hyeonseok Jeong, In-Uk Song, Yong-An Chung, Jong-Sik Park, Seung-Hee Na, Jooyeon Jamie Im, Marom Bikson, Wonhye Lee, Seung-Schik Yoo

**Affiliations:** 1 Department of Nuclear Medicine, Incheon St. Mary’s Hospital, College of Medicine, The Catholic University of Korea, Seoul 21431, Korea; hsjeong@catholic.ac.kr (H.J.); jooyeonim@gmail.com (J.J.I.); 2 Department of Radiology, Incheon St. Mary’s Hospital, College of Medicine, The Catholic University of Korea, Seoul 21431, Korea; 3 Department of Neurology, Incheon St. Mary’s Hospital, College of Medicine, The Catholic University of Korea, Seoul 21431, Korea; 77jjongsik@hanmail.net (J.-S.P.); seunghee.na@gmail.com (S.-H.N.); 4 Department of Biomedical Engineering, The City College of New York, New York, NY 10031, USA; bikson@ccny.cuny.edu; 5 Department of Radiology, Brigham and Women’s Hospital, Harvard Medical School, Boston, MA 02115, USA; wonhye.lee@gmail.com (W.L.); seungschik@gmail.com (S.-S.Y.)

**Keywords:** transcranial focused ultrasound, Alzheimer’s disease, regional cerebral metabolic rate of glucose, cognition, blood–brain barrier

## Abstract

Preclinical studies have suggested that low-intensity transcranial focused ultrasound (tFUS) may have therapeutic potential for Alzheimer’s disease (AD) by opening the blood–brain barrier (BBB), reducing amyloid pathology, and improving cognition. This study investigated the effects of tFUS on BBB opening, regional cerebral metabolic rate of glucose (rCMRglu), and cognitive function in AD patients. Eight patients with AD received image-guided tFUS to the right hippocampus immediately after intravenous injection of microbubble ultrasound contrast agents. Patients completed magnetic resonance imaging (MRI), ^18^F-fluoro-2-deoxyglucose positron emission tomography (PET), and cognitive assessments before and after the sonication. No evidence of transient BBB opening was found on T1 dynamic contrast-enhanced MRI. However, immediate recall (*p* = 0.03) and recognition memory (*p* = 0.02) were significantly improved on the verbal learning test. PET image analysis demonstrated increased rCMRglu in the right hippocampus (*p* = 0.001). In addition, increases of hippocampal rCMRglu were correlated with improvement in recognition memory (Spearman’s *ρ* = 0.77, *p* = 0.02). No adverse event was observed. Our results suggest that tFUS to the hippocampus of AD patients may improve rCMRglu of the target area and memory in the short term, even without BBB opening. Further larger sham-controlled trials with loger follow-up are warranted to evaluate the efficacy and safety of tFUS in patients with AD.

## 1. Introduction

Alzheimer’s disease (AD) is characterized by progressive neurodegeneration with cognitive and functional declines [1]. The major neuropathological manifestations are extracellular deposition of amyloid plaques and intracellular neurofibrillary tangles [1], and the hippocampus has been known to be one of the most affected brain structures in AD [2]. Despite the increasing prevalence of AD, currently available pharmacotherapies have modest benefits for symptom management and do not prevent neuronal loss and progressive cognitive deterioration [3]. Thus, novel treatment strategies such as noninvasive brain stimulation are under active investigation in recent years [3,4,5].

Low-intensity transcranial focused ultrasound (tFUS) applies acoustic energy to specific brain regions, with high spatial resolution and penetration depth [6]. It can excite or suppress neuronal activity without thermal damage, in addition to transiently opening the blood–brain barrier (BBB) in targeted areas when combined with microbubble ultrasound contrast agents (MB) [7]. It has been reported that tFUS-induced oscillations of MB may lead to amplification of local pressure waves and subsequent stretching of the endothelial walls [7].

Preclinical studies have suggested that BBB opening using tFUS can be used for efficient drug delivery in AD [8,9,10]. In mouse models of AD, anti-amyloid beta antibodies delivered to the brain after tFUS increased BBB permeability, resulting in reduced plaque pathology [9]. Moreover, BBB disruption alone reduced amyloid plaque burden and improved neural plasticity and spatial memory, independent of any therapeutic agents [11]. Recently, a few pilot studies reported the possibilities of tFUS-mediated BBB opening in small numbers of AD patients [12,13,14,15]. However, its efficacy and safety remain to be further established. In addition, changes in brain function and cognition induced by BBB opening are also unknown.

We investigated the effects of tFUS to the hippocampus on BBB disruption, the regional cerebral metabolic rate of glucose (rCMRglu), and cognitive function in patients with AD. For this ongoing research effort, our previous case report first described the results from the original four subjects, suggesting potential improvement of rCMRglu and cognition [16]. For this study, we added six additional test subjects to the cohort, such that the present analysis makes use of data from all ten test subjects—six new ones described here and the original four from our published report [16].

## 2. Materials and Methods

### 2.1. Study Design

**Figure 1:**
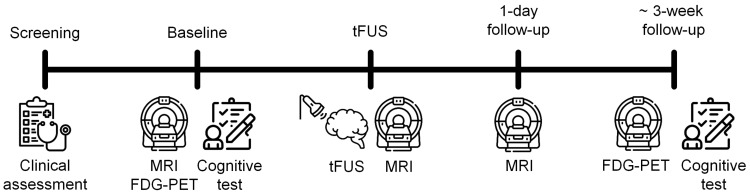
Study timeline. FDG–PET, ^18^F-fluoro-2-deoxyglucose positron emission tomography; MRI, magnetic resonance imaging; tFUS, transcranial focused ultrasound.

After baseline cognitive assessment and brain magnetic resonance imaging (MRI), computed tomography (CT), and positron emission tomography (PET) scans were performed, low-intensity tFUS was applied to the right hippocampus with intravenous injection of MB. Immediately and one day after the sonication, MRI scans were acquired to detect transient BBB opening and closing, respectively. Follow-up neuropsychological tests and PET scans were conducted within one month (about 20 days and 26 days, respectively) after the tFUS procedure. All patients were hospitalized one day before the tFUS procedure and discharged one day after the sonication for safety monitoring. In addition, patients were followed up in the outpatient clinic to assess delayed adverse events. The study timeline is presented in Figure 1.

### 2.2. Participants

Patients with probable AD were recruited from the neurology outpatient clinic at Incheon St. Mary’s Hospital (Incheon, South Korea). The clinical diagnosis of AD was determined based on the Diagnostic and Statistical Manual of Mental Disorders-IV criteria [17] and the National Institute of Neurological and Communicative Disorders and Stroke and the Alzheimer’s Disease and Related Disorders Association criteria [18]. The inclusion criteria were patients aged between 65 and 85 years; Mini-Mental State Examination (MMSE) < 20; Clinical Dementia Rating (CDR) ≥ 1. The exclusion criteria included neurological or psychiatric disorders other than AD; radiological findings on MRI such as hemorrhage, infarction, or tumor; contraindications to MB, MRI, or MRI contrast agents. This study was approved by the Institutional Review Board and registered on Clinical Research Information Service (KCT0005098). All patients or legal guardians gave written informed consent.

### 2.3. Clinical Assessment

The screening consisted of medical history, physical, and neurological evaluations, electrocardiography, chest X-ray examinations, routine blood test, and brain MRI and CT. Cognitive function was evaluated with MMSE, CDR, Digit Span Test, Seoul Verbal Learning Test (SVLT), Contrasting Program, Go/No-Go Test, Controlled Oral Word Association Test, and Color Word Stroop Test. The neuropsychological tests were performed by a neuropsychologist who was blinded to the study purpose.

### 2.4. Baseline Image Acquisition

For the baseline scans, fiducial markers that are visible in both MRI and CT images (PinPoint, Beekley Corp., Bristol, CT, USA) were attached to four locations of the patient’s head for image guidance of tFUS [19,20].

Brain MRI scans were conducted using a 3T MR scanner (MAGNETOM Skyra, Siemens, Enlargen, Germany) equipped with a 32-channel head coil. T1-weighted MPRAGE (repetition time (TR) = 2.000 ms; echo time (TE) = 2.49 ms; field of view (FOV) = 230 × 230 mm^2^; matrix = 256 × 256; flip angle = 9°; voxel size = 0.9 × 0.9 × 0.9 mm^3^), fluid-attenuated inversion recovery (FLAIR; TR = 9.000 ms; TE = 81 ms; inversion time = 2.500; FOV = 224 × 224 mm^2^; matrix = 256 × 320; flip angle = 90°; voxel size = 0.7 × 0.7 × 4.0 mm^3^), and susceptibility-weighted imaging (SWI; TR = 27 ms; TE = 20 ms; FOV = 203 × 224 mm^2^; matrix = 232 × 256; flip angle = 15°; voxel size = 0.44 × 0.44 × 1.50 mm^3^) scans were acquired.

Brain PET–CT images were obtained using a Discovery STE PET–CT scanner (GE Healthcare, Milwaukee, WI, USA). After intravenous injection of 185–222 MBq of ^18^F-fluoro-2-deoxyglucose (FDG), patients waited for approximately 45 min lying in a supine position with their eyes closed in a room with dimmed light. Transaxial PET (matrix = 128 × 128; pixel size = 1.95 × 1.95 mm^2^; slice thickness = 3.27 mm) and CT images (FOV = 250 × 250 mm^2^; matrix = 512 × 512; pixel size = 0.49 × 0.49 mm^2^; slice thickness = 0.50 mm) were acquired. PET images were reconstructed with standard filtering methods and the ordered subset expectation–maximization algorithm. No patients had significantly large calcifications within the cranial cavity on CT scans (> 3 mm) that may distort ultrasound propagation.

### 2.5. Application of tFUS

**Figure 2:**
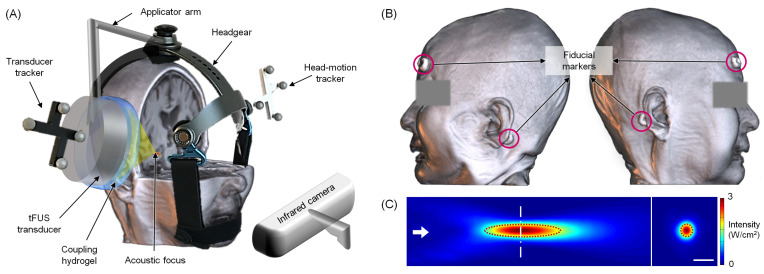
(**A**) Illustration of the experimental setup for transcranial focused ultrasound (tFUS) sonication to the right hippocampus of patients with Alzheimer’s disease. Image-guided real-time navigation was performed using the motion-tracking infrared camera which monitors the location and orientation of the headgear and the FUS transducer through optical trackers. The transducer is placed over the entry point on the scalp, and the coupling hydrogel is positioned between the transducer and the scalp. The sonication path is overlaid on the T1-weighted magnetic resonance imaging (MRI) of a patient. (**B**) A 3D visualization of four fiducial markers that were placed on the patient’s head during the acquisition of MRI and computed tomography (CT) scans. The markers were used as reference coordinates to co-register the patient’s virtual (MRI and CT) and real space for image-guided tFUS sonication. (**C**) The acoustic intensity profile of the FUS transducer along the sonication direction (the left panel; the sonication direction is depicted in an arrow) and from the transversal section at the focus at a centerline (shown in the right panel). The dotted red lines indicate the profile bound by the full-width at half-maximum of the peak intensity. Bar = 10 mm. The measurement was conducted in degassed water. The detailed methods were described in Jeong et al. 2021 [16].

An image-guided tFUS system (NS-US100, Neurosona Co., Ltd., Seoul, Korea) was used to apply tFUS to the right hippocampus. The experimental setup for tFUS sonication is illustrated in Figure 2. For the image guidance, T1-weighted MPRAGE and CT data were co-registered. After the patient was seated on a chair, the image space was registered to the physical space based on the spatial coordinates of the fiducial markers attached to the head under optical tracking (NDI, Ontario, Canada). A compressible cryogel (Bluemtech, Wonju, Korea) and ultrasound hydrogel (Aquasonic, Parker Laboratories, Fairfield, NJ, USA) were used for the sonication transducer. The sonication focus was targeted to the right hippocampus, while the sonication path was perpendicular to the skull and avoided the sinus and thick skull segments (> 10 mm). Immediately after the intravenous injection of MB (Definity, Lantheus Medical Imaging Inc., North Billerica, MA, USA; 10 μL/kg over one minute), tFUS was delivered using the following parameters (7): fundamental frequency = 250 kHz; tone-burst duration = 20 ms; pulse repetition frequency = 2 Hz; duty cycle = 4%; treatment duration = 180 sec; in situ mechanical index = 0.30–0.88.

### 2.6. Follow-Up Image Acquisition

After approximately 30 min from the sonication, follow-up MRI scans were conducted. In addition to FLAIR and SWI, T1-weighted images with two different flip angles (TR = 4.42 ms, TE = 2.01 ms, FOV = 224 × 224 mm^2^, matrix = 224 × 224, flip angle = 2° or 14°, voxel size = 1.0 × 1.0 × 3.0 mm^3^) were acquired. The 3D gradient-echo sequence (3D CAIPIRINHA-controlled aliasing in parallel imaging results in higher acceleration) was used to obtain T1 dynamic contrast-enhanced (DCE)–MRI images (TR = 3.32 ms; TE = 1.12 ms; 24 reference lines for both phase and partition encoding using an acceleration factor of 2 × 2 with reordering shift 1; FOV = 224 × 224 mm^2^; matrix = 224 × 224; flip angle = 9°; voxel size = 1.0 × 1.0 × 4.0 mm^3^; 48 volumes; total scan time = 10 min and 11 s) after intravenous injection of gadobutrol (Gadovist, Bayer Healthcare, Wayne, NJ, USA) using an automated injector pump at a dose of 0.1 mL/kg with a flow rate of 2.5 mL/s, followed by a 25 mL saline flush with a flow rate of 2.0 mL/s. The second follow-up MRI scans were conducted one day after the sonication using the same sequences. Follow-up FDG–PET scans were also performed with the same parameters as the baseline scans.

### 2.7. Image Analysis

Transient BBB opening and closing were assessed with visual inspections of contrast enhancement in DCE–MRI images. In addition, independent component analysis (ICA) was conducted for DCE–MRI data [21]. Using Statistical Parametric Mapping 12 (SPM; https://www.fil.ion.ucl.ac.uk/spm, accessed on 15 November 2021) and FMRIB Software Library (https://fsl.fmrib.ox.ac.uk/fsl/fslwiki, accessed on 15 November 2021), all volumes of DCE–MRI data were realigned, skull-stripped, and concatenated for each patient. The preprocessed individual DCE–MRI data were decomposed into 20 independent components (IC) using the Infomax algorithm in the Group ICA of fMRI Toolbox (GIFT; https://trendscenter.org/software/gift, accessed on 15 November 2021). The ICASSO toolbox repeated the ICA algorithm 20 times to assess the stability of the ICs. For each patient, ICs with initial signal increases, followed by continuing elevation, were visually checked.

SPM was also used for preprocessing and statistical analysis of FDG–PET data. For each patient, the PET scan was co-registered to the T1-weighted MPRAGE scan. After T1-weighted images were normalized to the Montreal Neurological Institute space, the transformation parameters were also applied to the co-registered PET data. Afterward, PET images were resliced to 2 mm isotropic resolution and smoothed using an 8 mm full-width at half-maximum Gaussian kernel. For each voxel, normalized rCMRglu was calculated as a ratio to global glucose metabolism using proportional scaling. A whole-brain voxel-wise paired *t*-test was performed to assess changes in rCMRglu. The statistical threshold was *p* < 0.005 with 30 or more voxels.

### 2.8. Statistical Analysis

Changes in cognitive measures were analyzed with Wilcoxon signed-rank test. Spearman’s rank correlations were calculated between significant changes of rCMRglu and those of cognitive outcomes. Statistical analysis was performed using Stata 17 (StataCorp., College Station, TX, USA).

## 3. Results

A total of 10 patients with probable AD were screened, and 2 patients were excluded from the study (one patient did not meet inclusion criteria, and one patient withdrew consent). Eight patients underwent application of tFUS to the right hippocampus and completed both baseline and follow-up assessments. Their demographic and clinical characteristics are presented in Table 1.

Radiological evidence of contrast enhancement associated with BBB opening was found in neither the visual inspection nor the ICA of the DCE–MRI data. No adverse events were observed during the hospitalization and follow-up outpatient visits for 5 to 24 months.

The neuropsychological test results are demonstrated in Table 2. The immediate recall (*z* = 2.21, *p* = 0.03) and recognition memory on the SVLT (*z* = 2.35, *p* = 0.02) were significantly improved after the sonication.

**Figure 3:**
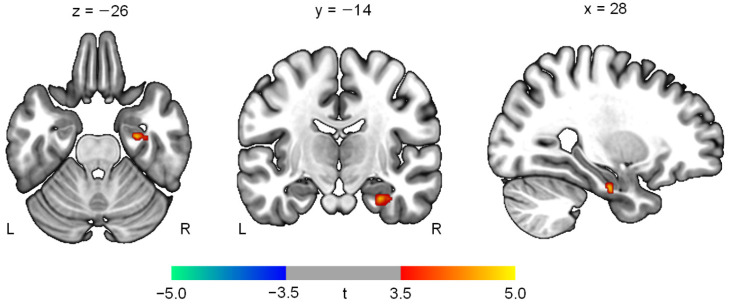
Changes in regional cerebral metabolic rate of glucose after transcranial focused ultrasound to the right hippocampus in patients with Alzheimer’s disease (*p* < 0.005). The numbers above the slices indicate Montreal Neurological Institute coordinates. The color bar represents *t* values at each voxel. L, left; R, right.

**Figure 4:**
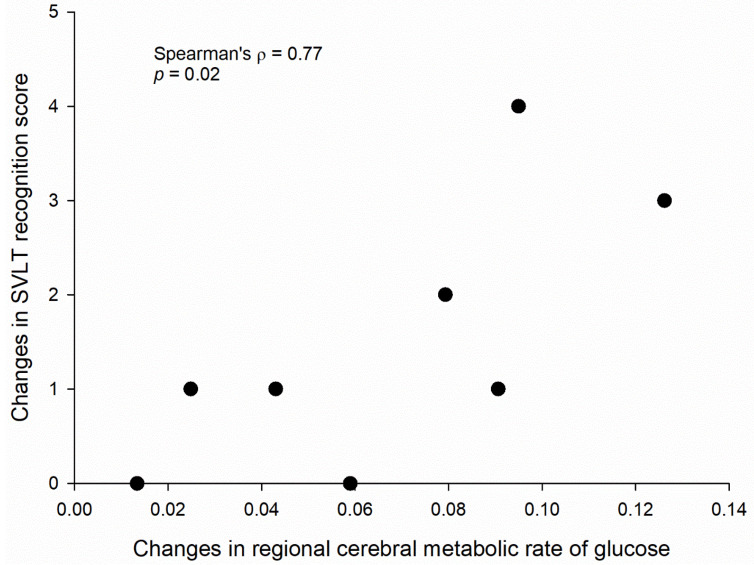
An association between changes in regional cerebral metabolic rate of glucose (rCMRglu) in the right hippocampus and changes of recognition memory on the Seoul Verbal Learning Test (SVLT) after transcranial focused ultrasound to the right hippocampus in patients with Alzheimer’s disease. Normalized rCMRglu was calculated as a ratio to global glucose metabolism.

The PET analysis showed an increased level of rCMRglu in the right hippocampus (*t* = 4.74; *z* = 3.07; *p* = 0.001; cluster size = 46 voxels; coordinates = 28, −14, −26) (Figure 3). Other brain regions, including the left hippocampus, did not demonstrate significant changes in rCMRglu. In addition, increases in hippocampal glucose metabolism were positively associated with the improvement of recognition memory (Spearman’s *ρ* = 0.77, *p* = 0.02) (Figure 4).

## 4. Discussion

This study investigated the effects of tFUS to the hippocampus on BBB opening, rCMRglu, and cognition in AD patients. Although the evidence of BBB opening was not observed on DCE–MRI, tFUS significantly enhanced hippocampal glucose metabolism and memory without adverse events. Furthermore, increases in rCMRglu in the hippocampus were correlated with improvement of recognition memory. Our results suggest that tFUS to the hippocampus may improve rCMRglu of the target area and memory in AD patients.

While we cautiously applied tFUS in AD patients due to the scarcity of previous human studies and potential adverse events such as microhemorrhage, the acoustic pressure level used in the current study may be insufficient to induce stable cavitation of MB and concurrent opening of the BBB. Consistent with this conjecture, a prior tFUS study reported that a mechanical index greater than 0.96 is required to enhance BBB permeability in ovine models when using the same type of MB [21]. Since successful BBB opening is dependent on various factors such as sonication parameters, types of MB, and transducer configurations, more studies are required to explore and optimize the tFUS protocols in humans.

Recently, there has been a growing interest in secondary effects of tFUS-mediated BBB opening. For instance, the inflammatory response decreases in amyloid-beta plaques and hyperphosphorylated tau proteins, and changes in regional cerebral blood flow and neural activity have been suggested [22]. In dementia rat models, tFUS-induced BBB disruption in the hippocampus increased brain-derived neurotrophic factor (BDNF), early growth response protein 1 (EGR1), and hippocampal neurogenesis, which lead to improved spatial memory [23]. Moreover, elevated levels of BDNF are maintained until 18 days after the BBB opening [23], indicating the prolonged effects on neurogenesis, synaptic plasticity, and membrane excitability. Although a transient and reversible reduction in glucose uptake was observed in normal rat brains immediately after BBB opening by tFUS [24], more long-term changes in rCMRglu remain unknown. In this study, the application of tFUS with MB did not open BBB in the hippocampus but significantly enhanced glucose metabolism of the target area. Our results suggest that MB cavitation at the subthreshold levels may also have secondary effects independent of detectable BBB opening and, therefore, increase hippocampal glucose metabolism.

Another possibility would be that tFUS per se increased hippocampal glucose metabolism, irrespective of BBB opening. It has been demonstrated that tFUS modulates structural and functional synaptic plasticity. For instance, tFUS alone stimulated neuronal activity and synchronous oscillations and increased BDNF expression in the mouse hippocampus [25]. Moreover, repeated tFUS to the rat hippocampus over 10 days enhanced density of dendritic spines, the expression level of glutamate receptor GluN2A subunit, and frequency of spontaneous excitatory postsynaptic current [26]. Decreased dendritic spine density and impaired maintenance of long-term potentiation have been reported in the hippocampus of AD animal models [27,28].

Although various mechanisms of tFUS have been suggested, intramembrane microcavitation may induce calcium influx and affect functions of ion channels and mechanoreceptors, thereby modulating neuronal excitability [29,30,31]. Several studies indicate that N-methyl-D-aspartate (NMDA) receptors show mechanosensitivity [32], and membrane tension can activate NMDA receptors by removing Mg^2+^ blocking of the channel [33]. Furthermore, increases of BDNF after tFUS upregulate expressions of GluN2A [34]. As the present study did not include a tFUS without MB group, we cannot determine whether the observed increases of hippocampal glucose metabolism reflect MB-induced effects or not.

Impairment in verbal learning and memory, one of the earliest clinical manifestations in AD, largely results from neuropathological deficits in the hippocampus and surrounding structures. In this study, the patients showed significantly better performances in immediate recall and recognition memory on the verbal learning test after the application of tFUS. Furthermore, a significant association was found between improvement in recognition memory performance and increases in glucose metabolism of the hippocampus. Previous FDG–PET studies reported that hippocampal hypometabolism in AD is linearly correlated with deficits in immediate recall and recognition [35,36]. Recognition memory consists of two components, recollection and familiarity, which may rely on the hippocampus and perirhinal cortex, respectively [37]. Although both processes are impaired in AD, recollection is more severely affected than familiarity [38]. Our results suggest that tFUS-induced enhancement of hippocampal glucose metabolism may have beneficial effects on memory function in AD patients.

Some limitations should be considered when interpreting our results. First, the lack of a sham control group precludes definitive conclusions regarding the effects of tFUS on rCMRglu and cognitive functions. A prior PET study reported that administration of placebo induced changes in rCMRglu in patients with depression, showing interactions with placebo effects on brain function [39]. In addition, potential practice effects on the repeated cognitive tests cannot be ruled out, although our sample consisted of patients with moderate-to-severe AD. Future studies should compare tFUS with sham treatment to evaluate the benefits of tFUS. Second, the sample size was small, and most of the patients had moderate-to-severe AD. Thus, studies including more patients with various severity are required to generalize our findings. Third, additional clinical evaluations such as amyloid PET and cerebrospinal fluid or plasma biomarkers would be useful to further characterize the study participants. Fourth, multiple comparison correction methods were not used in the statistical analyses due to the small sample size.

In conclusion, the application of low-intensity tFUS to the hippocampus with MB may enhance hippocampal glucose metabolism and memory function in the short term, even without BBB opening. With higher spatial resolution and penetration depth than other noninvasive neuromodulation techniques, tFUS may be a novel therapeutic option for AD. Our results are preliminary, and further larger sham-controlled trials are warranted to confirm the efficacy and safety of tFUS in patients with AD. In addition, longer follow-up of more than a month and assessments of dose-dependent responses of repeated tFUS will elucidate long-term benefits and facilitate clinical applications of tFUS in AD.

## Figures and Tables

**Table 1:** Demographic and clinical characteristics of the study participants.

Characteristics	Mean ± SD or *n*
Age (years)	78.1 ± 2.9
Sex (male/female)	1/7
Education (years)	9.9 ± 6.1
MMSE	5.63 ± 4.57
CDR	
1	1
2	6
3	1
CDR-SOB	12.63 ± 4.07

CDR, Clinical Dementia Rating; CDR-SOB, Clinical Dementia Rating-Sum of Boxes; MMSE, Mini-Mental State Examination.

**Table 2:** Changes in neuropsychological test results after transcranial focused ultrasound.

Test	Baseline (Mean ± SD)	Change (Mean ± SD)	Test ^a^
MMSE	5.63 ± 4.57	1.00 ± 1.51	*z* = 1.53, *p* = 0.13
Digit Span Test: forward	3.00 ± 2.00	0.13 ± 0.64	*z* = 0.58, *p* = 0.56
Digit Span Test: backward	0.50 ± 0.93	0.00 ± 0.00	
SVLT: immediate recall	0.88 ± 1.46	0.75 ± 0.71	*z* = 2.21, *p* = 0.03
SVLT: delayed recall	0.00 ± 0.00	0.00 ± 0.00	
SVLT: recognition	11.50 ± 0.53	1.50 ± 1.41	*z* = 2.35, *p* = 0.02
Contrasting Program	1.63 ± 3.54	3.00 ± 4.81	*z* = 1.72, *p* = 0.09
Go/No-Go Test	1.00 ± 2.83	1.63 ± 3.50	*z* = 0.74, *p* = 0.46
COWAT	0.88 ± 1.25	0.00 ± 1.07	
CWST: word reading	28.29 ± 41.77 ^b^	−5.83 ± 10.57 ^c^	*z* = −1.71, *p* = 0.09
CWST: color reading	1.71 ± 4.11 ^b^	−0.67 ± 1.63 ^c^	*z* = −1.00, *p* = 0.32

^a^ Wilcoxon signed-rank test. ^b^ *n* = 7. ^c^ *n* = 6; COWAT, Controlled Oral Word Association Test; CWST, Color Word Stroop Test; MMSE, Mini-Mental State Examination; SVLT, Seoul Verbal Learning Test.

## Data Availability

The datasets presented in this article are not readily available because the IRB has restrictions on sharing datasets. Requests to access the datasets should be directed to Y.-A.C. (yongan@catholic.ac.kr).
